# Efficacy of accelerated intermittent Theta-Burst Stimulation (aiTBS) in treatment-resistant depression: study protocol for a randomized controlled trial

**DOI:** 10.1192/j.eurpsy.2025.1300

**Published:** 2025-08-26

**Authors:** J. Andreo-Jover, R. Mazuecos, C. Rízea, J. Marin Lozano, E. Jiménez Solá, J. M. Pastor, P. Sánchez-Castro, M. F. Bravo Ortiz, R. Villanueva Peña

**Affiliations:** 1 Department of Psychiatry, Universidad Autonoma de Madrid; 2 Hospital La Paz Institute for Health Research (IdiPAZ); 3 Department of Psychiatry, Clinical Psychology and Mental Health; 4 Department of Clinical Neurophysiology, Neurology Service., La Paz University Hospital; 5 Universidad Autonoma de Madrid, Madrid, Spain

## Abstract

**Introduction:**

New antidepressant treatments are needed that not only demonstrate strong efficacy but also act quickly, are safe for long-term use, and are well-tolerated by patients. Accelerated intermittent Theta-Burst Stimulation (aiTBS) is a promising form of noninvasive brain stimulation in the treatment of depression (1). iTBS stimulation is a time saving and cost-effective technique, and could provide long-lasting improvement of treatment-resistant depression (TRD) (2). Currently, there is limited evidence regarding the role of aiTBS within treatment algorithms for TRD compared to placebo.

**Objectives:**

This is a single-center, double-blind, randomized, placebo-controlled pilot intervention study, designed to evaluate the efficacy and safety of aiTBS for TRD.

**Methods:**

Stimulation will be administered in 10 daily sessions, one session every 50 minutes, over 5 days, for a total of 50 sessions. Each session will consist of 3 minutes of stimulation at a frequency of 50 Hz, delivering 600 stimuli per session, with an intensity of 120% of the motor threshold targeted at the left dorsolateral prefrontal cortex.

**Results:**

Participants must be adults aged 18 to 75 years (inclusive), and meet the criteria for TRD, unresponsive to two antidepressants at an adequate dose and for an adequate duration with a stable dose for at least 4 weeks. Assessments, including HDRS, PHQ-9, EQ-5D-5L, SDS, CGI-S, and MGH-ATRQ, will be conducted at the baseline visit, at the end of the treatment, one and three months after the treatment.

**Conclusions:**

Accelerated protocols are an emerging area of interest in the treatment of TDR, but several issues remain to be clarified, including the durability of their effects, safety, and feasibility of implementing these protocols in outpatient settings in routine clinical practice.

**Disclosure of Interest:**

None Declared

